# Evaluating a Variable Porosity Wound Dressing With Anti-Scar Properties in a Porcine Model of Wound Healing

**Published:** 2018-05-24

**Authors:** Collynn F. Woeller, Aubrey Woodroof, Shannon H. Lacy, Patrick S. Cottler, Jane L. Gui, Angela Piñeros-Fernandez, Stephen J. Pollock, Richard P. Phipps

**Affiliations:** ^a^Department of Environmental Medicine, University of Rochester School of Medicine and Dentistry, Rochester, NY; ^b^PermeaDerm, Inc, Carlsbad, Calif; ^c^Department of Plastic Surgery, University of Virginia, Charlottesville; ^d^Department of Microbiology and Immunology, University of Rochester School of Medicine and Dentistry, Rochester, NY

**Keywords:** salinomycin, chronic wound, wound dressing, excessive scarring, myofibroblast

## Abstract

**Introduction:** New treatments that promote wound healing while preventing scar formation are needed. One option in topical wound healing is the use of temporary dressings that allow the natural healing process with minimal scar formation. **Methods:** We evaluated the temporary wound dressings PermeaDerm C, and a PermeaDerm C derivative coated with the anti-scarring agent, salinomycin (PermeaDerm D) in a pig model of wound healing to show the efficacy of these wound dressings in vivo. **Results:** Porcine fibroblasts grow well in the presence of PermeaDerm C or PermeaDerm A, and salinomycin reduces excessive myofibroblast formation in porcine fibroblasts in vitro. In vivo, wounds treated with PermeaDerm C and PermeaDerm A did not show abnormal or unwanted healing patterns up to 8 weeks post–wound formation. Wounds covered with either PermeaDerm C or PermeaDerm A showed a more mature wound-healing phenotype than the control wounds. **Conclusions:** PermeaDerm C and PermeaDerm A allowed wound healing, revealing the potential of both PermeaDerm C and PermeaDerm A to promote effective healing while preventing excessive scar formation.

Surface wound healing of skin is a process in which resident fibroblasts and other progenitor stem cells grow and repair damaged tissue areas. Often in cases of severe burns, chronic inflammation, or other persistent injury, healing can proceed out of control with the accumulation of excessive contractile myofibroblasts, the key effector cells that mediate scarring.^1^^,^^2^ Myofibroblasts highly express α-smooth muscle actin (αSMA), an important protein required for wound contraction, and myofibroblasts also produce large amounts of extracellular matrix material including collagen and fibronectin.^3^^-^5 Hypertrophic scars, which arise as a result of an excessive wound-healing response with an accumulation of myofibroblasts after skin injury, have been treated with a temporary dressing of occlusive silicone gel sheeting.^6^^-^8 While occlusive silicone sheeting has proven effective for treatment of hypertrophic scars, there have been few temporary dressings that have proved to be useful for healing of chronic wounds or severe burns. Silicone gel coating has been used as a treatment of burn scars and has shown efficacy in reducing several features of scarring including reduced vascularity, improved pigmentation, and increased pliability of the scarred area.^9^ Here, we test a novel temporary wound dressing called PermeaDerm C (PDC), a clinician-controlled, variable porosity, bilaminate matrix made of nylon mesh and silicone that contains gelatin and Aloe Vera extract (Immuno-10)^10^ for chronic wounds and PDC derivatives coated with the anti-scarring agent and natural antibiotic, salinomycin, termed PermeaDerm A (PDA), to promote wound healing while limiting excessive scar formation.^11^

## METHODS

### PDC and PDA production and cell culture

Temporary wound dressings were a gift from PermeaDerm Inc and produced as previously described.^10^^,^^11^ Briefly, medical-grade silicone with a nylon mesh was coated with gelatin and Immuno-10 (purified carbohydrate fraction of Aloe Vera) using precision spraying equipment. PDA had additional coating material that contained salinomycin (both in the silicone layer and in the biological coating [gelatin/Immuno-10 coating]). Dressing sterilization was performed by exposing PDC and PDA to 25 kGy of electron beam radiation. Cell culture was performed as previously described.^12^ Adult and neonatal porcine dermal fibroblasts were purchased from Cell Biologics, Inc (Chicago, Ill). Media and supplements were purchased from Gibco/Invitrogen (Carlsbad, Calif) and Cell Biologics, Inc.

### Western blot

Total protein was harvested from 2 × 10^6^ cells and lysed in 60 mM Tris, pH 6.8, 2% SDS, containing 1× protease inhibitor cocktail (Sigma, St Louis, Mo), and analyzed for αSMA (mouse anti-αSMA, cat. # A2547; Sigma-Aldrich) and β-tubulin (rabbit anti-β-tubulin, cat. # 2146; Cell Signaling Technology, Danvers, Mass) by Western blot as previously described.^13^ Anti-mouse or anti-rabbit horseradish peroxidase (HRP)–conjugated secondary antibodies were obtained from Jackson Immunoresearch (West Grove, Pa). Western blot signals were visualized using Immobilon Western chemiluminescent HRP substrate (Millipore, Billerica, Mass), with images captured using a c-DiGit Blot Scanner and Image Studio software (Li-Cor, Lincoln, Neb).

### In vivo testing of PDC and PDA

Animal experiments were performed under a protocol approved by the University of Virginia Institutional Animal Care and Use Committee (Animal Welfare Assurance #A3245-01) in accordance with the National Institutes of Health's Guide for the Care and Use of Laboratory Animals. Pigs were housed in an AAALAC-accredited facility. Two female domestic Yorkshire swine (35 kg) were used for the study. On the day of surgery, animals were anesthetized, intubated, and their skin shaved. The surgery area was cleansed with iodophor solution. Five 3 × 3-cm^2^ wounds were marked and tattooed on either side of the spine (wounds separated by at least 3 cm) for a total of 10 wounds. To control for animal growth, an additional 3 × 3-cm^2^ unwounded square was tattooed cranially to the wounds. Excisional wounds down to muscle fascia were created with a scalpel, following the markings, and complete hemostasis was achieved using pressure and electrocauterization. After achieving hemostasis, 3 × 3-cm^2^ samples of either PDC or PDA were placed into each wound bed.

Four wounds each were covered with PDC and PDA samples. The wounds were arrayed in an anterior to posterior fashion, with 4 wounds treated with PDA paired positionally and with 4 wounds treated with PDC. The two control wounds were created posteriorly to the 8 treated wounds. In addition, because of the size of the animals (length of torso) with respect to the wound size, all of the wounds were able to be placed in positions that were not directly adjacent to joint movement. The wound dressings were placed with the textured side in contact with the wound, with a strain of 10% in both axes to open up the porosity, and held in place with 4 interrupted sutures in the corners. The 9th and 10th wounds served as a control. Wounds were then packed with dry gauze as a bolster and circumferentially wrapped. Animals received postoperative analgesics and were continually monitored in a warmed environment until fully recovered from anesthesia. Animals were reanesthetized at weekly intervals (1, 2, 3, and 4) and at week 8 postwounding. At the defined time points, the gauze dressings were replaced without interfering with the PDC or PDA, and the wounds photographed to quantify contracture. At weeks 4 and 8, a biopsy down to the underlying muscle was taken from 2 wounds treated with PDC and 2 wounds treated with PDA, and 1 untreated control. At weeks 1, 2, and 3, all wounds were analyzed for contraction. We also measured the thickness of each wound when created, and there was not any statistical difference at any of the 5 longitudinal positions. Therefore, granulation tissue formation and epithelialization needed would be similar for all wounds.

Biopsy samples were fixed in 10% formalin, processed, and sliced for histological and immunohistological assessment to quantify wound healing. The wounds analyzed histologically were randomly selected. Paraffin-embedded slides were sectioned onto slides and stained with hematoxylin and eosin (H&E), Masson's trichrome, and Picrosirius red by Histoserv Inc (Germantown, Md). Stained slides were visualized and assed for wound healing and scar formation.

## RESULTS

### Salinomycin in PDA attenuates myofibroblast formation in porcine fibroblasts

**Figure 1:**
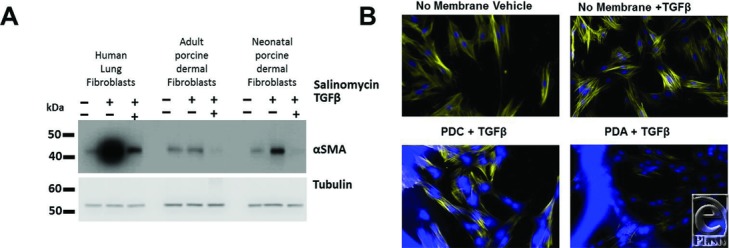
Salinomycin and PDA reduce myofibroblast formation. (a) Human lung fibroblasts and porcine adult and neonatal dermal fibroblasts were plated in 6-well culture dishes and treated with vehicle (DMSO), TGFβ (5 ng/mL), or TGFβ + salinomycin (250 nM) for 3 days. After 3-day incubation, cells were harvested and analyzed by Western blot for the myofibroblast marker, αSMA, and a loading control, β-tubulin. TGFβ treatment leads to a large induction of αSMA production in all 3 fibroblast types. Salinomycin treatment led to a dramatic reduction in αSMA production. (b) Neonatal porcine dermal fibroblasts were plated in 6-well culture dishes with or without PDC or PDA (salinomycin coated) in the presence of TGFβ (5 ng/mL) for 5 days. Cells were then fixed and stained with AlexaFluor 594–conjugated phalloidin (yellow, stains actin filaments) and DAPI (blue, DNA stain that shows cell nucleus). Cells were analyzed by fluorescence imaging using an EVOS-FL Digital Imager. TGFβ increased the number and intensity of actin filaments, indicating the formation of filamentous and contractual myofibroblasts. PDA reduced the number of actin filaments, demonstrating an attenuation of the myofibroblast phenotype. PDA indicates PermeaDerm A; αSMA, alpha-smooth muscle actin.

Using a high-throughput screen of natural products and existing drugs, we discovered that salinomycin is a potent anti-scarring agent.^12^ While salinomycin works effectively in human fibroblasts, its anti-scarring use has not been tested in porcine cells. As we ultimately desired to evaluate salinomycin in a pig model of wound healing, we tested its efficacy to block TGFβ-driven myofibroblast formation in porcine-derived fibroblasts. Here, human lung fibroblasts (control), adult porcine dermal fibroblasts, or neonatal porcine dermal fibroblasts were treated with vehicle (DMSO), TGFβ (5 ng/mL), or TGFβ plus 250 nM salinomycin for 72 hours to allow myofibroblast formation. Cells were then harvested and analyzed for the myofibroblast marker, αSMA and tubulin (loading control), by Western blot (Fig 1*a*). TGFβ treatment resulted in a robust induction of αSMA, especially in human lung fibroblasts and neonatal porcine fibroblasts. A smaller induction was observed in the adult porcine dermal fibroblasts. Importantly, in all fibroblast strains tested, salinomycin dramatically reduced αSMA expression.

Since porcine dermal fibroblasts responded well to the anti-scarring activity of salinomycin, we evaluated whether or not pig fibroblasts grow well and respond to the temporary dressings PDC and PDC coated with salinomycin (termed PDA). Neonatal porcine fibroblasts were grown in the presence or absence of PDC or PDA for 5 days (Fig 1*b*). Both PDC and PDA allowed growth of porcine dermal fibroblasts. Interestingly, PDA reduced excessive filamentous actin stress fiber formation and the myofibroblast phenotype as demonstrated by a reduction in the number of visible actin filaments (Fig 1*b*, bottom right). Taken together, these studies reveal that porcine fibroblasts grow well in the presence of either temporary wound dressing and that salinomycin reduces excessive myofibroblast formation.

### In vivo wound healing with PDC and PDA

**Figure 2:**
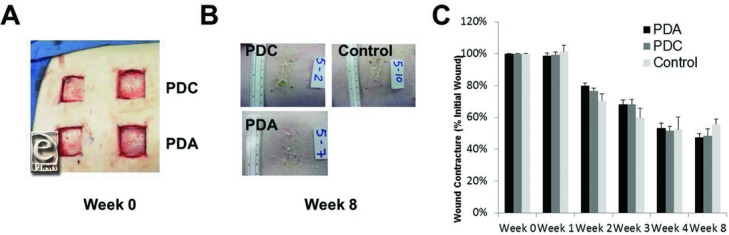
PDC and PDA allow wound contracture and healing in a pig model. (a) Representative images of wound sites on animals immediately after wound formation. Sections of both PDC and PDA were used to cover the wound sites. (b) Representative wound sites at 8 weeks after wound formation. (c) Wound contracture data based on original wound size at 100%. The wounds in each of the 3 groups did not demonstrate any immediate contraction by week 1. By the second week, wound contraction led to wound sizes as a percentage of initial of 70.6% ± 4.4% in control wounds and 76.1% ± 3.4% and 76.6% ± 3.0% for wounds treated with PDA and PDC, respectively. Wound contracture is complete around 4 weeks, with wound sizes of 50.1% ± 3.3%, 48.4% ± 3.2%, and 52.4% ± 7.8% for PDA-, PDC-, and control-treated wounds, respectively. PDC indicates PermeaDerm C; PDA, PermeaDerm A.

Since we observed that porcine fibroblasts could grow well in the presence of PDC and PDA and salinomycin reduced the expression of the myofibroblast marker αSMA in porcine fibroblasts, we performed a pilot in vivo study using PDC and PDA as temporary wound dressings in a porcine model of wound healing. Two female domestic Yorkshire swine were used for the preliminary study. Each animal received 10 uniform wounds. The temporary dressings PDC and PDA were applied to 8 wounds (4 per animal) each, and 4 control wounds (2 per animal) received gauze dressing. All 20 wounds were assessed for contracture at weeks 0, 4, and 8 (see representative images at weeks 0 and 8; Figs 2*a* and 2*b*). The wounds in each of the 2 groups did not demonstrate any immediate contraction in the first week (Fig 2*c*). By the second week, wound contraction led to wound sizes as a percentage of initial of 70.6% ± 4.4% in control wounds and 76.1% ± 3.4% and 76.6% ± 3.0% for wounds treated with PDA or PDC, respectively. Consistent with previous reports,^14^ wound contracture is complete around 4 weeks, with wounds sizes of 50.1% ± 3.3%, 48.4% ± 3.2%, and 52.4% ± 7.8% for PDA-, PDC-, and control-treated wounds, respectively (Fig 2*c*). There were not any statistical differences seen among the 3 groups at any of the discrete time points.

The Patient and Observer Scar Assessment Scale has been a validated as a reliable scoring system to evaluate scars.^15^ The system scores the scar parameters of vascularity, pigmentation, thickness, relief, pliability, surface area, and overall opinion in a scale of 1 (normal skin) to 10 (worst scar imaginable). Here, wounds were still healing and scars were too immature to score prior to the 8-week time point when reepithelialization was complete. Only the observer portion of the scale was used to evaluate 4 wounds treated with PDC and PDA and 2 control wounds. Three trained and blinded observers scored the wounds. The scores for each of the parameters were less than 5, indicating the scars were healing without major complications. There were no significant differences among the treatment groups for any of the parameters, indicating that wound healing and contracture could take place in the presence of PDC or PDA. In addition, there was not any difference in vascularity, pigmentation, thickness, relief, pliability, surface area, and overall for any of the 3 groups. Analysis was performed on Minitab using an analysis of variance and Tukey's post hoc multiple comparisons (*P* < .05).

**Figure 3:**
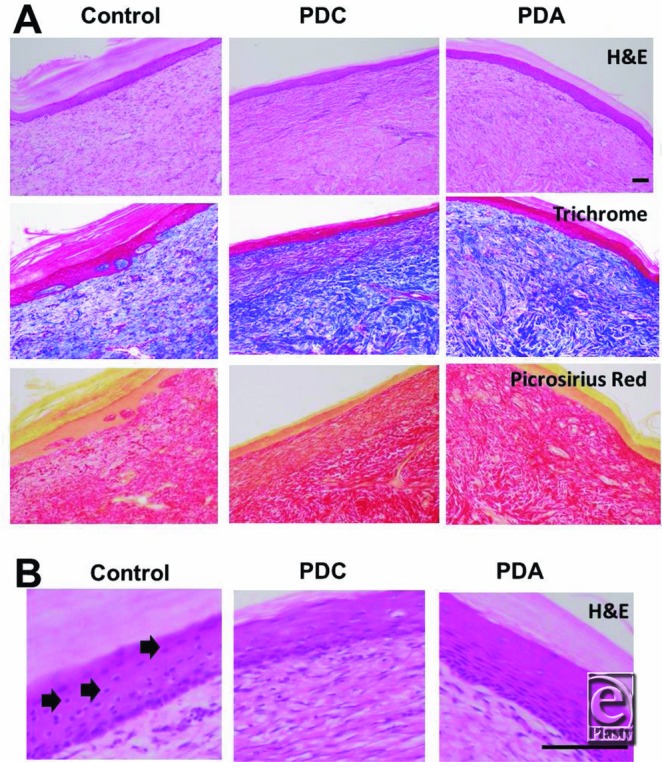
Histological assessment of control and PDC- and PDA-covered wounds in a pilot pig model. (a) Representative histological analysis (H&E, trichrome, and Picrosirius red) of wound sites at 8 weeks. Representative images of biopsies from control wounds or wounds covered with PDC and PDA are shown. (b) Enlarged H&E images show the cellularity of the epidermis. Black arrows denote increased cellularity of the control wound compared with PDC- and PDA-covered wounds, especially in the region closest to the stratum corneum. Images were captured on an Olympus BX51 microscope with an original magnification of 100×. Bar indicates 100 μm. PDC indicates PermeaDerm C; PDA, PermeaDerm A; and H&E, hematoxylin and eosin.

Biopsy samples from control wounds and wounds covered with PDC or PDA were fixed, processed, and sliced for histological assessment to monitor wound healing. Paraffin-embedded slides were sectioned onto slides and stained with H&E, Masson's trichrome, and Picrosirius red to observe wound healing and collagen network formation. Representative images from control and PDC- and PDA-covered wounds are shown in Figure 3.

All wounds analyzed by histology, as visualized by H&E, trichrome, and Picrosirius red staining, showed a normal reepithelialization 8 weeks after wounding. Importantly, all wounds treated with the temporary wound dressings PDC and PDA did not show abnormal or unwanted healing patterns up to 8 weeks post–wound formation. Interestingly, wounds covered with either PDC and PDA showed a more mature wound-healing phenotype than the control wounds. As observed in Figure 3, the control wound shows more cellularity in the epidermis (especially closer to the stratum corneum) than PDC- and PDA-covered wounds (black arrows, Fig 3*b*). In addition, from the Picrosirius red staining, the PDC- and PDA-covered wounds show a more mature collagen network than the control wound (bottom row, Fig 3*a*). These results reveal that both PDC and PDA allow normal wound healing of open wounds and may promote formation of more mature wound healing than control treated wounds.

## DISCUSSION

The ability of both PDC and PDA to allow normal wound healing in an in vivo model reveals the efficacy of these temporary wound dressings. Importantly, there were no significant differences in vascularity, pigmentation, or thickness of the healing wound for any of the 3 groups in this study (PDA, PDC, and gauze dressing wounds). These findings support the concept that PDA and PDC do not impair wound closure or healing. In addition, wounds treated with PDC or PDA appear to have a more mature wound-healing phenotype as demonstrated by a more mature collagen network and a lower epidermal cell number than control wounds that were treated with gauze bandaging. Occlusive or semiocclusive silicone sheeting has been shown to be an effective treatment option to reduce scarring in hypertrophic scars.^6^ In future studies, it may be interesting to compare the ability of the variable porosity temporary wound dressings used in this study with occlusive or semiocclusive silicone sheeting. A major difference between PermeaDerm and occlusive silicone sheeting is that PermeaDerm allows fluid exchange during wound healing without the need to replace the dressing.

In addition, in this pilot study, we did not observe any unwanted side effects in wounds treated with either temporary wound dressing. This result is important, but given our small sample size (8 wounds per temporary dressing on 2 animals), this should be followed up with additional studies using a larger sample size. We choose the porcine model of wound healing for this first study because of the physiologically similarities between human and porcine cutaneous wound healing.^16^^,^^17^ For example, porcine cutaneous wound healing, like human wound healing, occurs primarily by reepithelialization whereas in the rodent, wound contraction is the primary healing mechanism.^18^ In addition, humans and pigs show similar thickness in the epidermis and dermis compared with rodents, which have much thinner skin. These and other physiological similarities of the porcine model to human wound healing pointed us to use this model for our initial study. However, given the cost and logistical burdens of porcine models, a limited number of animals can be used. A larger study with more subjects used and with additional time points will be advantageous.

Importantly, salinomycin incorporation into PDA may have the added benefit of inhibiting microbial growth within the wound. Salinomycin, a natural antibiotic produced by *Streptomyces albus*, has potent antibacterial activity against gram-positive bacteria.^19^ Thus, while salinomycin is incorporated into PDA for anti-scarring properties,^12^ it may have additional antimicrobial activity as well. Further tests aimed at anti-scarring and antimicrobial activities of PDA and other PermeaDerm derivatives will help elucidate these effects.

We did not observe a statistically different contracture or wound-healing response with PDC as opposed to PDA; however, this may be expected, given the duration of the study and the nature of the wound. An 8-week in vivo wound-healing model allows skin to heal with proper wound closure but does not mimic a chronic wound or hypertrophic scar response. Future studies or trials looking at chronic wounds or severe burns may reveal the beneficial anti-scar effects of PDA as opposed to PDC. Both PDC and PDA contain a biological coating of gelatin and Immuno-10. Gelatin, an irreversibly hydrolyzed form of collagen, and Immuno-10 (a carbohydrate-rich extract from Aloe Vera) may have additional effects on wound healing and scar formation that need to be further characterized. The 4 main phases of wound healing (hemostasis, inflammation, proliferation, and maturation) may be altered by the presence of gelatin, Immuno-10, and salinomycin. While salinomycin can prevent proliferation of myofibroblasts, gelatin and Immuno-10 may be important in limiting the inflammatory phase of wound healing.^20^ Future studies aimed at understanding the mechanisms of action of these components will provide important insight into their function.

## CONCLUSION

Both PDC and PDA allowed wound healing in an in vivo porcine model. These results suggest that PDC and PDA can serve as temporary wound dressings for the treatment of chronic wounds.
